# Core temperature measurement methods and their accuracy in pediatric intensive care units: a narrative review

**DOI:** 10.3389/fped.2026.1800625

**Published:** 2026-04-01

**Authors:** Huilian Chen, Hongyuan Huang, Junjie Ning, Yinhui Li, Lina Qiao

**Affiliations:** 1 Pediatric Intensive Care Unit, West China Second University Hospital, Sichuan University, Chengdu, China; 2 NHC Key Laboratory of Chronobiology (Sichuan University), Chengdu, China

**Keywords:** accuracy, core temperature, hemodynamic instability, infrared thermography, non-invasive monitoring, pediatric intensive care, thermometry, zero-heat-flux

## Abstract

**Background:**

Accurate core temperature monitoring in Pediatric Intensive Care Units (PICUs) is crucial for assessing health status, guiding therapies, and predicting outcomes.

**Objective:**

To review core temperature measurement methods and their accuracy in pediatric intensive care units (PICUs), with emphasis on emerging non-invasive technologies.

**Content:**

We compare commonly used peripheral, non-invasive, and invasive techniques (including temporal artery, tympanic, zero-heat-flux thermometry, infrared thermography, and esophageal/rectal/bladder/nasopharyngeal sites) and summarize factors that affect agreement with core temperature under critical illness.

**Conclusion:**

Accurate temperature assessment supports timely diagnosis and therapy guidance. Evidence to date suggests that zero-heat-flux thermometry and infrared thermography are promising for continuous, non-invasive monitoring, but require further validation across pediatric ages and clinical scenarios.

## Introduction

In contemporary medical practice, body temperature monitoring is a cornerstone of clinical care, particularly in PICUs (1–4). The underdeveloped physiological systems and rapid metabolic changes of pediatric patients—such as fluctuations in oxygen consumption and energy expenditure—demand precise and reliable monitoring to facilitate the timely recognition of critical illness. Despite its importance, standardized protocols for core temperature monitoring remain insufficient (5).

The immature thermoregulatory mechanisms of pediatric patients render them highly vulnerable to environmental influences (2). Abnormal temperatures can lead to rapid clinical deterioration (6), and significant fluctuations caused by high metabolic rates may obscure signs of clinical decline, such as occult sepsis. For example, preterm infants and neonates have severely limited thermoregulatory capabilities, with hypothermia being directly linked to increased mortality rates (7). Moreover, studies indicate that deviations in core temperature exceeding 1 °C during therapeutic hypothermia are significantly associated with adverse neurological outcomes (8). Therefore, continuous and accurate core temperature monitoring is crucial for evaluating pediatric patients’ health status, aiding diagnosis, assessing therapeutic efficacy, and predicting clinical outcomes.

Since the late 19th century, body temperature has been regarded as a key vital sign in clinical care. Core temperature—defined as the temperature of the blood perfusing the hypothalamus—is a key indicator of the body's thermal balance and is vital for maintaining homeostasis and physiological function (1, 9). Abnormal core temperatures can impair enzyme activity, disrupt neural function, and adversely affect other processes (1), making accurate measurement in the PICU critically important.

However, there is still no universally accepted standard technique for determining core temperature (10, 11). Suitable methods may vary with patient age; for instance, neonates with thin skin and immature thermoregulation require particularly precise yet safe approaches. In critically ill children, pronounced temperature fluctuations necessitate continuous monitoring, but repeated rectal measurements are often impractical due to discomfort and slow response times. Measurement accuracy can also be compromised by factors like device calibration and operator technique—for example, an improper angle when using a tympanic thermometer. Furthermore, in the PICU, peripheral vasoconstriction (often due to vasopressor therapy) reduces skin blood flow, causing surface temperatures to significantly underestimate core temperature (12). Relying solely on surface measurements can therefore compromise clinical assessment.

In PICU practice, core or core-proxy temperature monitoring is particularly warranted when (i) hemodynamic instability or shock is present, (ii) active cooling/rewarming or tight temperature control is required, (iii) rapid temperature transitions are expected, or (iv) key clinical decisions depend on small temperature differences (e.g., escalation of vasoactive therapy or sepsis evaluation) (3, 5).

Currently, the most precise methods involve contact with well-perfused core sites like the esophagus, bladder, or pulmonary artery, where temperatures are stable and reflective of true core temperature (10). However, as these are invasive, they are not routinely used and should be reserved for cases where the benefits outweigh the risks.

This review evaluates the accuracy, practicality, and safety of different core temperature measurement methods for pediatric patients and aims to provide guidance for future research and clinical practice improvements.

## Thermoregulation

Thermoregulation is a complex physiological process involving multiple mechanisms to maintain a stable body temperature. The regulation of surface temperature is crucial for preserving core temperature stability in response to ambient fluctuations (2). This is achieved primarily by modulating cutaneous blood flow and sweat gland activity via hypothalamic control through autonomic pathways, including sympathetic adrenergic responses (12).

Children, particularly neonates and infants, have an immature thermoregulatory center. Their compensatory responses—such as shivering and brown fat activation—are underdeveloped, rendering them less efficient in responding to temperature changes and more vulnerable to environmental influences. The difference between core and surface temperature is influenced by multiple factors, including ambient conditions, measurement site, thermoregulatory capacity, and stress levels (13–15).

Hyperthermia and fever are not interchangeable. Fever reflects a regulated upward shift in the thermoregulatory set-point, whereas hyperthermia typically represents unregulated heat accumulation or impaired heat dissipation. In critically ill children, sustained high temperatures may increase metabolic demand and oxygen consumption, potentially worsening cardiorespiratory stress; thus, interpreting whether a measured elevation reflects fever vs. hyperthermia is clinically important (2, 3, 12).

Hypothermia in children may arise from environmental exposure, impaired heat production, anesthetic or sedative effects, or shock-related hypoperfusion. Clinically, hypothermia can be associated with coagulopathy, arrhythmias, and impaired immune responses. These physiologic changes can also amplify peripheral-to-core temperature gradients, making surface-based measurements particularly unreliable during low-perfusion states (2, 11).

Ambient temperature is a key determinant of the absolute core-to-surface temperature gradient. Lower ambient temperatures decrease surface temperature and challenge thermoregulatory capacity; however, if thermoregulation remains intact, core temperature can still be maintained within a normal range (16–18).

In the PICU, critically ill children often exhibit compromised thermoregulation due to factors such as peripheral vasoconstriction or vasopressor administration. These conditions can impair physiological responses like sweating and vasomotion, making surface temperature an unreliable proxy for core temperature. Therefore, direct and accurate measurement of core temperature is essential for precise clinical assessment (12).

Despite this, rapid and direct core temperature measurement is not always feasible in clinical practice. Given its convenience, surface temperature retains significant practical value as an accessible, indirect indicator of thermal status, aiding in the ongoing evaluation of critically ill pediatric patients.

## Peripheral body temperature

In the PICU, critically ill children often experience compromised thermoregulation due to peripheral vasoconstriction or vasopressor administration. This impairs physiological responses such as sweating and vasomotion, rendering surface temperature an unreliable indicator of core temperature. Therefore, accurate core temperature measurement is essential for precise clinical assessment. However, direct core temperature measurement is not always rapidly feasible in practice. Consequently, due to its ease of acquisition, body surface temperature retains significant practical value as an indirect indicator, aiding in the evaluation of a patient's thermal status.

Common PICU interventions further influence measurement agreement. Vasoactive agents and vasoconstrictors reduce cutaneous perfusion and increase peripheral-to-core gradients, causing skin- and axillary-based readings to underestimate core temperature during shock or low cardiac output states (12). Sedation, analgesia, and neuromuscular blockade may blunt behavioral and physiologic thermoregulatory responses (e.g., shivering), complicating interpretation of peripheral readings and potentially delaying recognition of clinically significant hypothermia or fever (3, 4, 19).

Since most thermoreceptors are located superficially, body surface temperature strongly correlates with thermal sensation (20). It is typically measured using thermometers or thermal imaging cameras, including axillary, forehead infrared, and skin patch sensors. These methods can help evaluate thermal comfort, stress, and thermoregulatory capacity under specific disease conditions (21). Although not a direct reflection of core temperature, surface temperature provides valuable insights into circulatory status and heat loss. For instance, skin temperature may serve as a reliable parameter for monitoring changes in cardiac output (22). In pediatric infectious disease ICUs, it has been demonstrated as a useful non-invasive indicator of peripheral hemodynamics in severe infections, aiding in the assessment of critically ill children (23). Some studies suggest that a peripheral-to-core temperature gradient (e.g., foot/skin temperature minus rectal temperature) exceeding 2 °C is a sensitive indicator (82% sensitivity) of early septic shock in children, highlighting the clinical relevance of the peripheral-core temperature gradient in pediatric sepsis (24).

As the body's largest organ, the skin accounts for up to approximately 90% of heat loss under normal conditions, though this proportion varies in critically ill patients depending on perfusion status (11). The discrepancy between skin and core temperature can be significant, particularly in critically ill children with impaired perfusion. Relying solely on skin temperature may therefore lead to inaccurate clinical judgments. It is essential to recognize both the utility and the limitations of surface thermometry across different clinical scenarios.

### Non-core peripheral measurements (axillary, oral, skin/forehead infrared)

A nationwide survey of 425 Chinese ICUs revealed that axillary temperature measurement remains the most prevalent method (96.47%), primarily due to its perceived safety and patient comfort (25). While an axillary temperature over 37 °C is often regarded as indicative of fever (26), this method is substantially influenced by the environment, particularly in patients exposed to cold, hypothermia, or low cardiac output states, where it may fail to accurately reflect core temperature.

Halabi et al., in a prospective study of preterm infants (<32 weeks gestational age), found a strong correlation between axillary and rectal temperatures; however, the variability between the two was more pronounced in hypothermic infants (27). This suggests that axillary measurement should be used cautiously in hypothermic infants, and alternative methods should be considered whenever feasible for more accurate monitoring.

Forehead (temporal artery) infrared thermometry, while convenient, can be influenced by ambient temperature or sweat. Sublingual (oral) temperature, though within a body cavity, is considered a secondary core temperature site. Its accuracy can be compromised by respiration, recent oral intake, and insufficient measurement time (requiring the mouth to be closed for over 3 min), placing its value between core and surface temperatures.

A PICU study comparing oral and axillary temperatures in orally intubated pediatric patients found oral measurement to be a feasible alternative. However, the measurement tools themselves can introduce error. Another study comparing electronic thermometers and single-use chemical dot thermometers in orally intubated patients showed a strong correlation (*r* = 0.937), but the chemical dot thermometers had significant inaccuracies: 11.8% of readings overestimated temperature, while 10.8% underestimated it by ≥0.4 °C (28). Therefore, in critical PICU settings where accurate temperature guides treatment, the use of chemical dot thermometers for oral monitoring is not advisable. Furthermore, oral measurement is less feasible in intubated or unconscious patients, further limiting its utility in the PICU.

## Core temperature measurement

### Non-invasive core temperature measurement

Non-invasive core temperature measurement has emerged as a significant research direction in the field of medical monitoring in recent years. Its aim is to accurately determine the temperature of deep body tissues, namely core temperature, using non-invasive methods, thereby addressing the invasiveness and complexity associated with traditional invasive measurements, such as rectal and esophageal temperature monitoring (10).

### Temporal artery temperature monitoring

Temporal artery thermometry estimates temperature from infrared emissions over the forehead/temporal region, seeking the peak signal over the superficial temporal artery and adjacent tissues (29). Pediatric studies report variable agreement between temporal artery readings and core-proxy sites, with performance influenced by age, ambient conditions, probe technique, and peripheral perfusion. Accordingly, temporal artery measurements should be interpreted cautiously in hemodynamically unstable children or during vasoconstriction (27). One study reported age-dependent differences when comparing temporal artery and bladder temperatures: among critically ill children aged 1–12 years, the mean difference was approximately 0.1 °C, suggesting close agreement in that subgroup (30). Where available, quantitative agreement metrics are summarized in Table 1.

**Table 1:** Agreement metrics across thermometry modalities.

Modality	Typical use in PICU	Common reference standard	Agreement (bias/LOA or key error)	Key limitations/notes
Axillary	Screening; common nursing measure	Rectal/core-proxy	Correlation with rectal reported; variability greater in hypothermic infants (19)	Affected by ambient temperature and perfusion; may underestimate in shock (17–19)
Oral	Selected non-intubated patients	Nasopharyngeal or core-proxy	Chemical dot: 11.8% overestimated; 10.8% underestimated by ≥0.4 °C (20)	Limited in intubated/unconscious; influenced by breathing/oral intake
Temporal artery	Rapid non-invasive spot check	Bladder/core-proxy	Mean difference vs. bladder ∼0.1 °C (age 1–12 years subgroup) (22, 29–31)	Technique/ambient/perfusion sensitive; caution with vasoconstriction (23)
Tympanic	Non-invasive repeatable	Rectal/bladder/nasopharyngeal	In low-perfusion states, the difference from bladder temperature may reach up to 1.2 °C; accuracy is also influenced by probe positioning and local anatomy (32–35)	Cerumen/narrow canal; requires correct probe angle
ZHF (SpotOn)	Continuous non-invasive core estimation	Bladder/rectal/esophageal	Bias vs. bladder −0.07 °C; LOA [−0.54, 0.40] °C; vs. rectal −0.24 °C; LOA [−0.81, 0.33] °C (36)	Sensor placement; may read slightly higher than esophageal (37)
IRT	Non-contact thermal distribution	Varies; inner canthus suggested	Feasibility demonstrated; validation studies needed (38–41)	Strongly affected by environment; core estimation not standardized
Rectal	Core-proxy intermittent/continuous	Varies	Lag during rapid changes; used as core-proxy (42)	Slow response; discomfort; contraindications
Bladder	Core-proxy continuous when catheterized	Intravascular/PAC (adult)	Used as core-proxy; close agreement with ZHF in study (27, 43–45)	Requires catheter; affected by urine flow
Esophageal	Core-proxy continuous (intubated/OR)	Intravascular/PAC (adult)	SpotOn slightly higher; mean diff 0.07 °C (28)	Probe depth/positioning; mainly intubated patients
Nasopharyngeal	Core-proxy continuous	Core-proxy/intravascular	Highest accuracy among four non-invasive methods in comparison study (46, 47)	Placement depth; nasal pathology/bleeding risk
Intravascular/PAC	Reference standard (mostly adults)	—	Gold standard for continuous core temp (48, 49)	Highly invasive; rare in PICU

Bias and limits of agreement (LOA) are reported when available; values vary by reference standard, setting, and patient physiology.

Vasoactive drugs are theoretically a significant factor that can influence temporal artery thermometry, as they induce peripheral vasoconstriction, which may alter local temperature readings (31). In a prospective observational study of critically ill adult patients, it was found that there was a significant difference between temporal artery temperature measurements and bladder temperature measurements. Specifically, in patients receiving norepinephrine, the temporal artery temperature measurements exhibited even greater deviations from the reference bladder temperature measurements (31). Therefore, in such cases, temporal artery thermometry may exhibit reduced accuracy when applied to critically ill patients. Temporal artery temperature measurement holds certain potential applications in PICUs. However, further research is required to validate its applicability and accuracy across diverse clinical settings. Simultaneously, it is essential to recognize the factors that may influence the accuracy of temporal artery temperature measurement and implement appropriate corrective actions when necessary.

### Zero-heat-flux (ZHF) thermometry

ZHF thermometry, a technique that minimizes heat loss through insulated sensors, measures subdermal temperatures at a depth of 1–2 cm to approximate core temperature values (50, 51). The ZHF thermometer enables clinicians to continuously and relatively non-invasively monitor body temperature, and its use is increasingly being adopted in intensive care settings as an alternative to more invasive core temperature measurements.

The SpotOn system, which utilizes zero heat flux technology, offers a non-invasive approach for continuous core temperature monitoring in critically ill patients. Studies in children have demonstrated that the temperature measured by SpotOn is slightly higher than rectal temperature but exhibits a strong correlation, suggesting that it more closely approximates core temperature compared to rectal thermometry (46). A study analyzed data from 748 critically ill patients who underwent simultaneous monitoring using the SpotOn system, continuous rectal, or continuous bladder temperature measurements. The recorded temperatures during the study ranged from 36.6 °C to 39.9 °C. The mean difference between SpotOn and bladder thermometry was −0.07 °C (SD, 0.24 °C; 95% limits of agreement, ±0.47 °C [−0.54 °C, 0.40 °C]). The mean difference between SpotOn and rectal thermometry was −0.24 °C (SD, 0.29 °C; 95% limits of agreement, ±0.57 °C [−0.81 °C, 0.33 °C]). In both groups, most temperature differences between methods were within ±0.5 °C (96% for bladder and 85% for rectal) (36). Additionally, the temperatures obtained using the SpotOn system demonstrated a high correlation and agreement with esophageal temperatures, with SpotOn temperatures being slightly higher than esophageal temperatures (mean difference 0.07 °C, *P* = 0.0287) (37). Therefore, the ZHF thermometer can be regarded as a promising non-invasive alternative for continuous core temperature monitoring in critically ill patients, particularly when invasive methods are contraindicated or unavailable, making it suitable for ongoing patient temperature monitoring.

### Infrared thermography (IRT)

IRT is a non-invasive method for temperature measurement. IRT works by detecting infrared radiation emitted by objects and converting this into thermal images, which display the distribution of surface temperature. Since all objects with a temperature above absolute zero emit infrared radiation, this radiation can be detected by a sensor to measure the temperature of an object (32). IRT is a non-invasive method for monitoring temperature changes in critically ill children, particularly in cases of hemodynamic instability, and has been explored for thermal pattern analysis and peripheral perfusion assessment in pediatric intensive care settings (38–41). The International Organization for Standardization has published a protocol for estimating temperature using infrared cameras, recommending that the inner canthus of the eye is the only facial area suitable for non-contact temperature measurement. A study investigated the use of IRT as a method for analyzing temperature curves in critically ill children. Infrared sensors were employed to capture images under clinical conditions, and it was demonstrated that thermography can effectively estimate body temperature (38). IRT has demonstrated significant potential in pediatric intensive care owing to its non-invasiveness, rapidity, and remote monitoring capabilities. It can provide real-time images of body temperature distribution, which is highly valuable for assessing and monitoring various disease states (39, 40). The systematic review conducted by Stanley et al. in 2024 further investigated the applications of IRT in acute diseases and demonstrated that IRT can be utilized in emergency departments and intensive care units for adults, children, and neonates. The study emphasized that while the clinical applications of IRT are largely in the early stages of development, it possesses substantial value for temperature monitoring and clinical use in critically ill children. The authors highlighted the necessity for higher-quality diagnostic validation studies to further solidify its clinical utility (41).

### Tympanic temperature measurement

Tympanic membrane temperature measurement is regarded as an approximation of core body temperature because the tympanic membrane shares the same vascular system with the hypothalamus via the internal carotid artery, thereby sharing its blood supply. Muma et al. demonstrated a strong correlation between tympanic membrane temperature and rectal temperature in their study conducted in the pediatric emergency department (48). In their study, Sadian et al. evaluated the accuracy and precision of four non-invasive peripheral temperature measurement methods (oral, axillary, tympanic, and forehead) in comparison to nasopharyngeal thermometry. The results indicated that tympanic temperature measurement exhibited the highest accuracy among the four methods (49). Currently, infrared tympanic thermometers, as a minimally invasive, repeatable, and relatively non-invasive method, can accurately reflect core body temperature and serve as an alternative to bladder or rectal measurements for pediatric temperature monitoring (33). For example, for patients with neutropenia, invasive temperature measurement methods should be avoided whenever possible, and tympanic temperature measurement is the preferred option (34). However, when the ear canal is narrow or blocked by cerumen in children, the measurement error may be substantial. In addition, thermometer positioning is an important determinant of measurement accuracy. Yeoh et al. reported that temperatures measured in the vicinity of the tympanic membrane were influenced by probe position and local anatomy, highlighting the importance of correct placement when using tympanic thermometers as an estimate of core temperature (33). It is crucial to ensure that the probe is accurately directed at the tympanic membrane. Niven et al.’s controlled study demonstrated that in children with low perfusion, the difference between ear temperature and bladder catheter temperature can reach up to 1.2 °C, suggesting that it may not be suitable for children in shock (52). Tympanic thermometry requires careful patient selection. When necessary, it should be combined with other temperature measurement methods for verification.

### Other non-invasive measurement methods

In a study comparing the accuracy of wearable core temperature measurement systems with bladder and tympanic thermometers in the ICU, Ehlers et al. demonstrated that while the wearable system provided continuous, non-invasive temperature monitoring, its accuracy failed to meet clinically relevant acceptance criteria. Although the wearable system exhibited comparable precision to tympanic measurement in at least one patient subgroup, it showed limited accuracy in correctly identifying fever when compared with invasive temperature measurement (53). Therefore, further research is warranted to investigate the application of wearable core temperature measurement systems in the intensive care unit.

Using a non-invasive, simple, and continuous temperature monitoring system represents an ideal choice. Cutuli et al. demonstrated that in ICU patients, non-invasive peripheral temperature measurement methods generally exhibit lower accuracy compared to invasive intravascular temperature measurement. Specifically, in adult patients, axillary, tympanic infrared, and zero heat flux thermometers all yielded lower readings than intravascular temperature (54). In a study assessing the accuracy of non-invasive thermometers conducted in an adult intensive care unit, researchers compared the consistency of four different non-invasive thermometers with pulmonary artery catheter temperature. The study demonstrated that, relative to the pulmonary artery standard, the tympanic thermometer exhibited the closest consistency, whereas the temporal artery thermometer showed the poorest consistency. Deviations exceeding 0.5 °C from the standard were relatively prevalent across all non-invasive devices (55). Similar to adults, various methods exist for measuring body temperature in children. However, owing to the particularities of pediatric temperature measurement (such as the immature temperature regulation center and a large surface area-to-weight ratio), children may exhibit reduced responsiveness to non-invasive thermometers. When selecting non-invasive thermometers, it is essential to thoroughly consider their consistency with the gold standard (e.g., pulmonary artery catheter temperature) and recognize that substantial differences may exist between devices, even within the clinically acceptable error range.

### Invasive core temperature measurement

Invasive temperature monitoring entails partially or fully inserting a temperature measurement device into the body to measure the temperature of specific tissues, such as blood, nasopharyngeal, bladder, or esophageal temperature. These invasive methods generally exhibit stronger correlations with intracranial core temperature and can provide precise temperature data for critically ill patients. The 2020 ESICM consensus statement recommends that in PICU settings, children with hemodynamic instability undergo invasive core temperature monitoring, such as bladder catheter thermometry (5).

### Blood temperature measurement

Invasive thermometry, particularly intravascular temperature measurement, is widely regarded as the gold standard for core temperature measurement (56). The pulmonary artery catheter (PAC) is inserted via central venous access into the right side of the heart and advanced into the pulmonary artery. It is widely regarded as the gold standard for continuous core temperature monitoring and is currently the most accurate method for measuring core body temperature in clinical settings. The PAC is predominantly employed in intensive care and cardiac surgery patients for monitoring hemodynamic parameters, including cardiac output, pulmonary artery wedge pressure, and vascular resistance. A thermistor situated at the distal port of the PAC measures blood temperature (57). Owing to the invasiveness of pulmonary artery catheterization, it is not routinely utilized in ICUs, especially in PICUs, thus restricting its application. Other intravascular temperature measurement methods, such as intravascular heat exchange techniques, intravascular catheters, and fiber optic temperature sensors, have scarce data available regarding their application in pediatric intensive care units and will not be elaborated upon further in this context.

### Nasopharyngeal temperature measurement

Nasopharyngeal temperature is continuously monitored using a fine thermistor probe inserted into the nasopharynx. Studies demonstrate a strong correlation between nasopharyngeal and core temperatures (11). In the PICUs, nasopharyngeal temperature measurement serves as an invasive method for assessing core body temperature in patients, particularly those requiring continuous temperature monitoring, such as mechanically ventilated patients or individuals with contraindications to other core temperature measurement methods. Nasopharyngeal temperature measurement is a widely adopted invasive technique in surgical and adult intensive care settings and has also been investigated in neonatal and pediatric populations. A study involving 157 tracheally intubated pediatric patients undergoing non-cardiac surgery demonstrated that nasopharyngeal thermometers can accurately measure core temperature, provided the probe is inserted to an age-appropriate depth. The optimal positioning of nasopharyngeal temperature probes in infants and children has been studied extensively. For infants younger than 6 months, the optimal probe position ranges from 6 to 10 cm; for infants aged 7–12 months, it is 7–8 cm; for children aged 13–23 months, it is 7.5–12 cm; and for children aged 6 years and older, it is 10–12 cm. Except for the 2- to 5-year-old group, the 95% limits of agreement (LOA) were <0.5 °C across all age categories, with the LOA extending from −0.67 °C to 0.52 °C at 9 cm in the 2- to 5-year-old group. At the optimal position within each age range, the bias (average nasopharyngeal-to-esophageal temperature difference) was ≤0.1 °C (47). However, in mechanically ventilated patients with endotracheal tube leakage, nasopharyngeal temperature may decrease, potentially underestimating the true core temperature. Similar to tympanic temperature measurement, nasopharyngeal temperature may yield falsely low readings in patients with hemodynamic instability. Furthermore, inaccurate probe positioning or nasal obstruction can result in erroneous temperature measurements.

### Bladder temperature measurement

Bladder temperature has been demonstrated to exhibit excellent consistency with pulmonary artery temperature (43). The kidneys receive approximately 25% of the cardiac output; therefore, when urine flow rate is within the normal range, bladder temperature closely approximates core body temperature. Erickson concluded in a study of critically ill adult patients that rectal temperature correlates strongly with bladder temperature, and bladder temperature more closely reflects core temperature than rectal temperature (44). Given that urine output is routinely monitored in ICU patients, bladder temperature measurement is highly practical and does not cause additional discomfort to patients. Its use is becoming increasingly prevalent in intensive care units (43). Studies have shown that bladder temperature remains stable irrespective of urine flow rate (45, 58). However, bladder temperature measurement is limited to children with indwelling urinary catheters and may pose a risk of secondary infections. Therefore, it should be used cautiously and removed promptly when no longer necessary.

### Esophageal temperature measurement

Esophageal temperature is closely correlated with pulmonary artery temperature and represents a fundamental method for measuring body temperature in intubated patients (11). However, the accuracy of esophageal temperature measurement depends on the positioning of the esophageal temperature probe. The optimal insertion depth of esophageal temperature probes (ETPs) in children is strongly associated with their height. A study involving children aged 3–13 years proposed a formula for calculating the optimal insertion depth of ETPs based on a retrospective analysis of 181 children who underwent minimally invasive repair of pectus excavatum: “Height/5 + 5 (cm)” (59). Optimizing the insertion depth helps minimize individual variations in pediatric patients, reduce the influence of environmental factors, and enhance procedural safety, thereby improving the quality of temperature monitoring.

### Rectal temperature measurement

In critically ill patients, although rectal thermometry is a traditional method for measuring body temperature, the excellent insulating properties of the rectum can cause the recorded temperature to lag behind core temperature changes. A study involving 45 infants with temperatures exceeding 38.5 °C compared body temperature measurements using temporal artery and rectal thermometers at 60 and 90 min after medication administration. The results demonstrated that the decrease in temperature measured by the temporal artery thermometer was significantly greater than that measured by the rectal thermometer at both 60 and 90 min post-medication. This suggests that rectal temperature changes may lag behind those of temporal artery temperature during periods of rapid body temperature fluctuation (42). Moreover, in critically ill children, blood is shunted to vital organs, leading to reduced intestinal blood flow under conditions such as hypothermia or shock. This can result in a rectal temperature that is lower than the actual core body temperature. Given the critical condition of these children, timely and accurate monitoring of body temperature is essential. Therefore, rectal temperature measurement should be used cautiously in PICUs. Another limitation of this method is the risk of rectal perforation, particularly in comatose patients with rectal wall dilation and thinning due to transient autonomic nervous system paralysis. Consequently, in clinical practice, when rapid responsiveness to changes in body temperature is required, alternative methods such as esophageal thermometry or bladder temperature monitoring should be considered to obtain more accurate temperature data. Additionally, studies on adult critically ill patients have shown that patients with neutropenia (Grade 2) should avoid the use of rectal thermometers (60), as this invasive procedure may cause discomfort or complications, especially in immunocompromised patients.

## Clinical implications and practical recommendations in the PICU

Among hospitalized patients, body temperature serves as a clinically significant, readily available, and directly measurable parameter. For instance, the recovery rate of hypothermia following burn surgery has been shown to predict mortality (57). Children possess a larger body surface area-to-mass ratio, which increases their susceptibility to evaporative heat loss. Compared with adults, children exhibit an immature thermoregulatory system, higher metabolic rates, and often less subcutaneous fat, rendering them more vulnerable to hypothermia. Hypothermia can impair platelet function, fibrinolysis, and the coagulation cascade, potentially leading to coagulopathy and increased transfusion requirements. In a prospective study involving 545 PICU patients, univariate comparisons and multivariate analyses of their temperature trajectories revealed that both fever and hypothermia are independent predictors of adverse outcomes in PICU patients (47). A study was conducted to evaluate the impact of mean body temperature during the first 24 h after admission on the short-term prognosis of pediatric sepsis patients. This retrospective cohort study analyzed data from patients admitted to a tertiary care hospital in China between 2010 and 2018, with a total of 1,144 sepsis patients included. The results demonstrated a roughly “U”-shaped relationship between body temperature on the first day of admission and in-hospital mortality. Specifically, both hypothermia and hyperthermia on the first day of admission were associated with an increased risk of in-hospital mortality for pediatric sepsis patients (43). This underscores the critical importance of precise measurement and evaluation of body temperature. Furthermore, prospective studies in adults have utilized temperature trajectories to identify novel sepsis subphenotypes, demonstrating that abnormal body temperature can offer prognostic information for patients with infections and reflect their underlying immune status. This implies that monitoring temperature changes in sepsis patients may aid in identifying distinct disease subtypes, thereby providing a foundation for personalized treatment strategies (44, 58). Therefore, body temperature is a critical parameter for evaluating the health status of pediatric patients, diagnosing diseases, and monitoring treatment outcomes. As an essential vital sign, body temperature not only guides the assessment of a patient's condition but also informs treatment decisions, thereby playing a pivotal role in the monitoring and management of organ function.

### When temperature measurement changes management

In the PICU, small differences between peripheral and core temperature can influence diagnostic interpretation and trigger interventions (e.g., antipyretic therapy, active cooling, active rewarming, sepsis evaluation, and sedation/analgesia adjustments). Discrepancies are most clinically consequential during hemodynamic instability, hypothermia, and rapid temperature transitions, when peripheral perfusion and heat redistribution amplify measurement error.

### Scenario-based selection of temperature monitoring methods

Table 2 summarizes pragmatic recommendations for choosing a monitoring site and device according to common PICU scenarios. These suggestions emphasize accuracy and responsiveness, balanced against invasiveness, feasibility, and patient safety. For hemodynamically stable children requiring intermittent checks, tympanic (with proper technique) or temporal artery thermometry may be used, while unexpected values should be confirmed with a core site when clinical decisions are high-stakes.

**Table 2:** Scenario-based selection of temperature monitoring in the PICU.

PICU scenario	Preferred measurement (if available)	Practical notes/cautions
Hemodynamic instability or shock (vasoactive support, poor perfusion)	Intravascular blood temperature, esophageal (if intubated), or bladder (if urinary catheter present)	Avoid relying on axillary/temporal/skin readings; peripheral vasoconstriction can underestimate core temperature. Confirm unexpected fever/hypothermia with a core site before escalating therapy.
Induced or accidental hypothermia; active rewarming	Esophageal or intravascular; bladder as a secondary option	Prefer continuous core monitoring; ensure probe positioning and secure fixation. Rectal readings may lag during rapid rewarming.
Rapid temperature shifts (post-resuscitation, severe infection, transfusion reaction)	Esophageal (intubated) or intravascular; ZHF as a non-invasive adjunct	Prioritize methods with fast response. If using ZHF, allow equilibration time and monitor for skin irritation under the sensor.
Post-operative cardiac surgery/deep sedation	Esophageal or bladder	Core monitoring is often already feasible due to intubation and catheterization. Be mindful of warmed humidified gases and probe depth.
ECMO/CRRT/advanced extracorporeal support	Circuit/blood temperature plus a core site (esophageal or bladder)	Circuit temperature reflects heat exchange and may differ from patient core temperature. Interpret trends alongside a patient core site and clinical status.

### Implementation tips and quality indicators

To improve reliability, PICUs should standardize (i) the default method for routine screening, (ii) triggers for confirming with a core site, and (iii) documentation of site, device model, and measurement conditions. Process indicators may include time to confirm abnormal readings, proportion of high-stakes decisions supported by core measurements, and device-related adverse events (e.g., skin injury with sensors, mucosal irritation with probes).

## Conclusion

The critical importance of temperature monitoring in the PICU cannot be overstated, as it is integral not only to the assessment of pediatric patients’ health status but also to disease diagnosis, treatment, and prognosis. Although no universally accepted standard technique for body temperature measurement has yet been established, the analysis presented in this article underscores the necessity of selecting an appropriate method based on clinical context to enhance the accuracy of temperature monitoring and the scientific rigor of clinical decision-making. With advancements in medical technology, body temperature measurement techniques continue to evolve. We recommend prioritizing ZHF thermometry and IRT for hemodynamically unstable children in PICUs, while reserving invasive methods (e.g., bladder catheters) for cases requiring gold-standard accuracy. Future guidelines should incorporate age-specific protocols for nasopharyngeal probe placement. Furthermore, future research should focus on validating and refining the accuracy and reliability of various temperature measurement methods, particularly their applicability across diverse clinical scenarios. Meanwhile, with the integration of big data and artificial intelligence technologies, body temperature monitoring is poised to become more intelligent and personalized. By leveraging large-scale temperature datasets, advanced predictive models can be developed to provide more precise and actionable insights for clinical practice.

In summary, although there are current challenges in body temperature measurement technology, the ongoing development of new technologies and in-depth research gives us confidence that future core temperature measurement will become more accurate, convenient, and personalized. This advancement will consequently provide higher-quality medical care for pediatric patients in PICUs.
